# Supplementary material for: Direct Observations of Amyloid β Self-Assembly in Live Cells Provide Insights into Differences in the Kinetics of Aβ(1–40) and Aβ(1–42) Aggregation
Source: Chem Biol. 2014 Jun 19;21(6):732–42. doi: 10.1016/j.chembiol.2014.03.014 (PMC4067742; doi:10.1016/j.chembiol.2014.03.014)
Supplement: Document S1. Supplemental Figures S1–S4 and Tables S1 and S2 [file mmc1.pdf]

Chemistry & Biology, Volume *21*

Supplemental Information

Direct Observations of Amyloid β Self-Assembly in Live Cells Provide Insights into Differences in the Kinetics of Aβ(1–40) and Aβ(1–42) Aggregation

Elin K. Esbjörner, Fiona Chan, Eric Rees, Miklos Erdelyi, Leila M. Luheshi, Carlos W. Bertoncini, Clemens F. Kaminski, Christopher M. Dobson, and Gabriele S. Kaminski Schierle

I. Supplementary figures

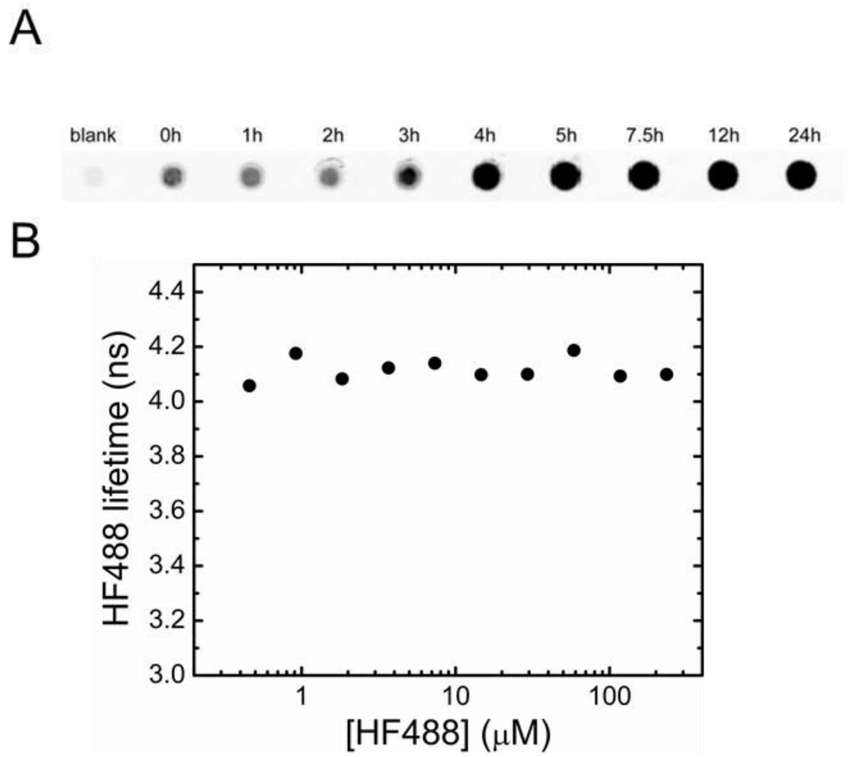

Figure S1 (related to Figure 1). *Fibril formation of HF488-labelled Aβ(1-42) peptides and fluorescence lifetimes of the HF488 dye in vitro.*

(A) Dot blot analysis of LOC immunoreactive fibrillar amyloid species in a solution of HF488-Aβ(1-42) peptide in 50 mM sodium phosphate buffer (pH 7.4) incubated in shaking condition (300 rpm orbital shaking) at ambient temperature. A densiometric analysis of this blot is shown in Fig. 1A of the main text. (B) Fluorescence lifetime of the unconjugated HF488 dye as a function of dye concentration. Note the logarithmic scale on the X-axis. The fluorescence lifetimes were measured on the FLIM microscope described in the experimental section but with the detection operated in single mode, which corresponds to a bulk measurement. The HF488 dye was suspended in 50 mM phosphate buffer, pH 7.4. The graph shows that the fluorescence lifetimes remain constant over at least two orders of magnitude higher concentration than what was used in this study to treat live cells. This shows that the HF488 fluorescence lifetime will not change merely due to the increased concentration of peptide accumulation within the cell.

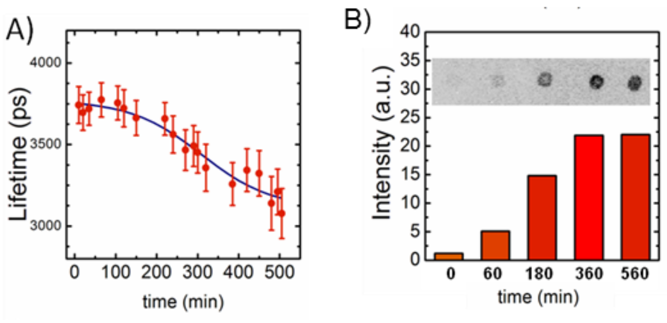

Figure S2 (related to Figure 2). *Fibril formation of HF488-labelled Aβ(1-40) in vitro.*

(A) Average fluorescence lifetime (±SD) as a function of time in an aggregating sample containing 5 µM HF488-Aβ(1-40) in 50 mM sodium phosphate buffer (pH 7.4). The data were extracted from an extended number of images from the series shown in Figure 2 of the main text. The sigmoidal curve is a fit to data and intended to guide the eye. (B) Dot blot analysis of the appearance of LOC immunoreactive fibrillar amyloid species in a sample of HF488-Aβ(1-40) peptide in 50 mM sodium phosphate buffer (pH 7.4) together with densiometric analysis of the blot showing the progressive increase in fibrillar species as a function of time.

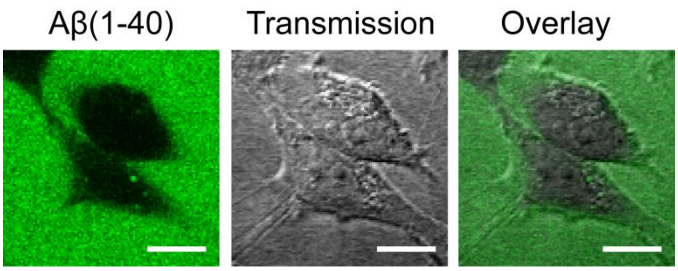

Figure S3 (related to Figure 3). *Cellular uptake of ^{HF488}Aβ(1-40) after 10 minutes of incubation.*

Fluorescence (left panel), transmission (middle panel) and overlay (right panel) confocal images showing SH-SY5Y cells treated with 500 nM ^{HF488}Aβ(1-40) for 10 minutes. The punctuate staining indicated vesicular uptake. Z-scanning was performed to ensure that the fluorescence emanated from peptide localised within the cell body. The scale bars are 10 µm.

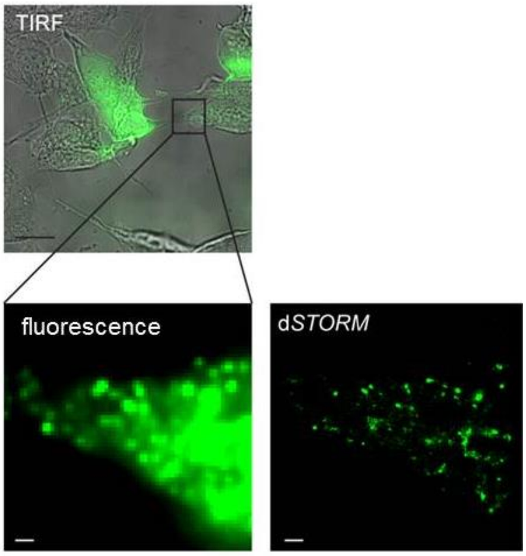

Figure S4 (related to Figure 5). *Improvement in resolution from direct stochastic optical reconstruction microscopy (dSTORM).*

Top panel: Total internal reflection (TIRF) image of a cell containing ^{HF647}Aβ(1-42). The image was acquired in highly inclined mode to access an image plane from within the cell. The zoom in depicts the area in the sample over which images for *d*STORM were acquired. Lower panels: The left panel shows the unresolved fluorescence image of the zoom in area depicted in the TIRF image and the right panel the resolved *d*STORM image. The scale bars in all images are 1 µm.

II. Supplementary Tables

Table S1 (related to Figure 4). Statistical analysis of the difference of the mean value of the intracellular lifetime of ^{HF488}Aβ(1-40) and ^{HF488}Aβ(1-42) compared to the extracellular lifetime. N denotes number of independent observations at each time point. P-values were computed in GraphPad Prism using the Fisher's Least Significant Difference (LSD) test (* p<0.05; ** p<0.01; *** p<0.001; **** p<0.0001).

	^{HF488}Aβ(1-40)			^{HF488}Aβ(1-42)		
	N	P Value	Significant	N	P Value	Significant
E.C. - 1h	2	0.55	ns	2	0.24	ns
E.C. - 3h	3	0.98	ns	3	0.12	ns
E.C. - 6h	3	0.98	ns	3	0.0028	**
E.C. - 24h	7	0.076	ns	6	< 0.0001	****
E.C. - 48h	9	0.0032	****	8	< 0.0001	****

Table S2 (related to Figure 4). Statistical analysis of the difference in intracellular lifetime between ^{HF488}Aβ(1-40) and ^{HF488}Aβ(1-42) at indicated time points. P-values were computed in GraphPad Prism using the Fisher's Least Significant Difference (LSD) test (* p<0.05; ** p<0.01; *** p<0.001; **** p<0.0001).

	^{HF488}Aβ(1-40) vs ^{HF488}Aβ(1-42)	
	P Value	Significant
0h	0.47	
1h	0.96	ns
3h	0.37	ns
6h	0.017	*
24h	0.0007	***
48h	0.22	ns
