# Supplementary material for: Direct Observations of Amyloid β Self-Assembly in Live Cells Provide Insights into Differences in the Kinetics of Aβ(1–40) and Aβ(1–42) Aggregation
Source: Chem Biol. 2014 Jun 19;21(6):732–42. doi: 10.1016/j.chembiol.2014.03.014 (PMC4067742; doi:10.1016/j.chembiol.2014.03.014)
Supplement: Document S2. Article plus Supplemental Information [file mmc2.pdf]

Cell Press

Chemistry & Biology
Article

Direct Observations of Amyloid β Self-Assembly in Live Cells Provide Insights into Differences in the Kinetics of Aβ(1–40) and Aβ(1–42) Aggregation

Elin K. Esbjörner,[1,2,*] Fiona Chan,[3] Eric Rees,[3] Miklos Erdelyi,[3] Leila M. Luheshi,[1] Carlos W. Bertoncini,[4] Clemens F. Kaminski,[3] Christopher M. Dobson,[1] and Gabriele S. Kaminski Schierle[3,*]
[1]Department of Chemistry, University of Cambridge, Lensfield Road, Cambridge CB2 1EW, UK
[2]Department of Chemical and Biological Engineering, Division of Chemistry and Biochemistry, Chalmers University of Technology, Kemivägen 10, 41296 Gothenburg, Sweden
[3]Department of Chemical Engineering and Biotechnology, University of Cambridge, New Museums Site, Pembroke Street, Cambridge CB2 3RA, UK
[4]Laboratory of Molecular Biophysics, Institute for Research in Biomedicine, Baldiri Reixac 10-12, 08028 Barcelona, Spain
*Correspondence: eline@chalmers.se (E.K.E.), gsk20@cam.ac.uk (G.S.K.S.)
http://dx.doi.org/10.1016/j.chembiol.2014.03.014
This is an open access article under the CC BY license (http://creativecommons.org/licenses/by/3.0/).

SUMMARY

Insight into how amyloid β (Aβ) aggregation occurs in vivo is vital for understanding the molecular pathways that underlie Alzheimer's disease and requires new techniques that provide detailed kinetic and mechanistic information. Using noninvasive fluorescence lifetime recordings, we imaged the formation of Aβ(1–40) and Aβ(1–42) aggregates in live cells. For both peptides, the cellular uptake via endocytosis is rapid and spontaneous. They are then retained in lysosomes, where their accumulation leads to aggregation. The kinetics of Aβ(1–42) aggregation are considerably faster than those of Aβ(1–40) and, unlike those of the latter peptide, show no detectable lag phase. We used superresolution fluorescence imaging to examine the resulting aggregates and could observe compact amyloid structures, likely because of spatial confinement within cellular compartments. Taken together, these findings provide clues as to how Aβ aggregation may occur within neurons.

INTRODUCTION

The misfolding and aberrant self-assembly of specific proteins into extracellular deposits or intracellular inclusions are the pathological characteristics of a range of severe and debilitating neurodegenerative disorders (Chiti and Dobson, 2006). In particular, the amyloid β peptide (Aβ) has been identified as a central and causative constituent of the pathology of Alzheimer's disease (AD), the most prevalent form of adult dementia (Hardy and Allsop, 1991). Aβ exists in several isoforms of which the 40-residue variant, Aβ(1–40), is the most abundant variant in vivo, whereas the more aggregation-prone, 42-residue variant, Aβ(1–42), is the major proteinaceous component of the extracellular senile plaques that are the hallmarks of this disease (Glenner and Wong, 1984; Masters et al., 1985).

The phenomenon of Aβ fibril formation has been studied extensively in vitro (Jarrett et al., 1993; Lomakin et al., 1996), and the recent development of sensitive fluorescence-based methods has shown that it is also becoming possible to trace in some detail the population of intermediate states (Lee et al., 2011; Narayan et al., 2012). Recent progress in the analysis of the kinetics of fibril formation has, in addition, revealed details of the microscopic assembly processes that underlie complex protein aggregation reactions, including those of Aβ, allowing for a mechanistic understanding of protein aggregation behavior under well defined and carefully controlled conditions in vitro (Cohen et al., 2013; Cohen et al., 2012). It is, therefore, of considerable interest and importance to find a way to provide detailed descriptions of the nature of the amyloid species formed during the aggregation of the Aβ peptide in vivo and to monitor directly in situ the kinetics of Aβ assembly. Such information should enable us to gain mechanistic insights into the processes by which Aβ aggregates are generated and how they proliferate in their specific biological contexts.

Most observations of Aβ aggregates formed in cells or tissue rely on indirect or invasive techniques, such as the collection of secreted Aβ oligomers in cell culture media (Walsh et al., 2000), biochemical analysis of high-molecular-weight aggregates in cell lysates (Hu et al., 2009), or immunohistochemical staining of aggregates in fixed samples (Capetillo-Zarate et al., 2011; Takahashi et al., 2004). Such approaches have revealed important results, showing, for example, that Aβ aggregates can, at least under certain circumstances, occur within neurons as well as in extracellular deposits. Additional methods are, however, needed to continuously monitor the kinetics of aggregation at the level of detail that is required to describe and understand the mechanistic steps involved in the self-assembly reactions that result in Aβ amyloid formation in vivo.

To address the nature and dynamics of the process of Aβ assembly in neurons, we took advantage of recently developed procedures that enable the conversion of proteins into amyloid fibrils to be monitored with great sensitivity by following the changes in the fluorescence lifetime of covalently linked dye labels. Such lifetimes have been shown to correlate directly with the appearance of β sheet-rich amyloid structures (Kaminski

Schierle et al., 2011a) and, thereby, to act as reporters of their development within a given sample. This approach makes it possible to use fluorescence lifetime imaging (FLIM) to monitor amyloid formation and, thus, complements previously devised, two-color fluorescence resonance energy transfer imaging methods for in vivo amyloid studies (Klucken et al., 2006; Roberti et al., 2007). We have used this technique in previous studies to image in real time, with the optical resolution of a confocal microscope, the formation of amyloid aggregates of α-synuclein and Tau in vitro from protein in solution and in vivo using a *C. elegans* model of Parkinson's disease (Kaminski Schierle et al., 2011a; Michel et al., 2014).

In this study we utilized this methodology to study the kinetics of aggregation of Aβ(1–40) and Aβ(1–42), the two most common isoforms of the Aβ peptide, during their cellular uptake and subsequent vesicular trafficking within live neuronal cells. A particular objective has been to gain insights into the phenomenon of accumulation of intraneuronal Aβ species. Intraneuronal Aβ has been observed in the brains of AD patients (Gouras et al., 2000) as well as in mouse models of AD (Billings et al., 2005) before the appearance of extracellular plaques. It is possible that at least some of this intraneuronal Aβ has been taken up from extracellular Aβ pools, especially in light of the observation that fluorescently labeled Aβ injected into the tail veins of mice with a compromised blood-brain barrier accumulates in neurons in the cerebral cortex (Clifford et al., 2007).

We also used the FLIM methodology in combination with superresolution imaging techniques (Kaminski Schierle et al., 2011b; Pinotsi et al., 2014) to relate the observed fluorescence lifetime to the physical size and shape of the Aβ aggregates in situ. The results show how the uptake of Aβ peptides from the external environment promotes the formation of Aβ aggregates within neuronal cells and identifies features in the in vivo aggregation kinetics of Aβ(1–40) and Aβ(1–42) that are valuable in developing an understanding of the mechanistic details of how intracellular Aβ may contribute to AD pathogenesis.

RESULTS

The Dependence of the Fluorescence Lifetime of the Hilyte Fluor 488 Dye on the Amyloidogenic State of the Aβ Peptide

Fluorescence microscopy is a powerful method for the detection of protein inclusions in cells by using fluorescence intensity as an indicator of aggregate formation (Kaganovich et al., 2008), although more advanced readouts are required to obtain information on the structural characteristics of the aggregation process. Indeed, more information can be obtained by measuring the fluorescence lifetime because this parameter has been found to be sensitive to the structural transition from disordered states into cross-β amyloid fibrils, specifically as a consequence of the changes in the fluorescence lifetimes as aggregation proceeds (Chan et al., 2013; Ghukasyan et al., 2010; Kaminski Schierle et al., 2011a; Michel et al., 2014; Pinotsi et al., 2013).

We first examined whether a Hilyte Fluor 488 (HF488) dye attached to the N termini of Aβ(1–40) and Aβ(1–42) could act as a reporter of amyloid formation by monitoring how the fluorescence intensity of the dye changes as fibril formation occurs in vitro for each of the two Aβ isoforms. The appearance of fibrillar species was identified by means of dot blot experiments using the conformation-specific antibody LOC (Kayed et al., 2007), and the HF488 fluorescence intensity was measured in parallel (Figure 1A; Figure S1 available online). Significant fluorescence quenching was observed to take place on a similar time scale as that of the appearance of fibrillar species, although the decrease in fluorescence intensity was somewhat slower than the increase in LOC immunoreactivity. This small difference, however, could result from the fact that LOC recognizes fibrillar oligomers as well as amyloid fibrils, whereas the fluorescence quenching is likely to be associated predominately with amyloid fibrils. We also confirmed the formation of β sheet structure by measurement of circular dichroism spectra at the beginning and end of the time course of the experiment (Figure 1B). Next, we used FLIM to analyze these samples collected at the beginning and end of the time course of the experiment. The fluorescence lifetimes in each pixel of these images were obtained by fitting monoexponential decay functions to the observed time-resolved fluorescence decays, and the data and images are shown in Figures 1C and 1D. The mean fluorescence lifetime for solutions containing fibrillar ^{HF488}Aβ(1–42) (3.3 ± 0.1 ns) was observed to be substantially lower than those containing monomeric ^{HF488}Aβ(1–42) (3.7 ± 0.05 ns), whereas larger aggregates of ^{HF488}Aβ(1–42) (as well as of ^{HF488}Aβ(1–40)) that were observed to deposit on the coverslip had even shorter lifetimes, often in the range of 2.5–3.0 ns. Similar results were obtained for ^{HF488}Aβ(1–40) (Figure 2; Figure S2), indicating that there are no inherent differences between the two Aβ isoforms in the context of the fluorescence lifetime changes exhibited by the HF488 reporter dye during aggregation. The results show that FLIM readings enable the detection of aggregated forms of HF488-labeled Aβ peptides and can distinguish these species from the corresponding labeled monomers.

For the HF488 dye to function as a direct reporter of amyloid formation in vivo, its fluorescence lifetime must respond to the conformational changes associated with amyloid formation but remain largely unaffected by extrinsic factors, such as the solution conditions that the Aβ peptides may encounter in the living cell. Therefore, we examined the response of the HF488 dye itself to a change in pH from 7.4 to 5.0 and found that the dye has a lifetime of 4.0 ± 0.1 ns in both cases (Figure 1E). That is, it is insensitive to pH changes in this range. We also found that its fluorescence lifetime is constant, within experimental error, across a wide range of dye concentrations (Figure S1B). This result shows that the intracellular accumulation of Aβ peptides would not, in the absence of aggregation, result in a reduction of fluorescence lifetime as a consequence of self-quenching interactions. Furthermore, the fluorescence lifetime of the unconjugated HF488 dye (4.0 ± 0.1 ns) is longer than that of the Aβ-conjugated dye (3.7 ± 0.05 ns), perhaps as a result of interactions between the dye and the polypeptide chain in the latter case (Chen et al., 2010). This finding suggests, therefore, that it may be possible to restore the fluorescence lifetime of HF488 by proteolytic degradation of the Aβ peptide. We tested this idea by treating ^{HF488}Aβ(1–40) with trypsin, a procedure that results in the cleavage of the Aβ peptide into short fragments. This treatment resulted in an increase in the mean fluorescence lifetime from 3.7 ± 0.1 ns to 4.0 ± 0.1 ns (Figure 1E), indicating that, unless the fluorescence lifetime of Aβ is observed to

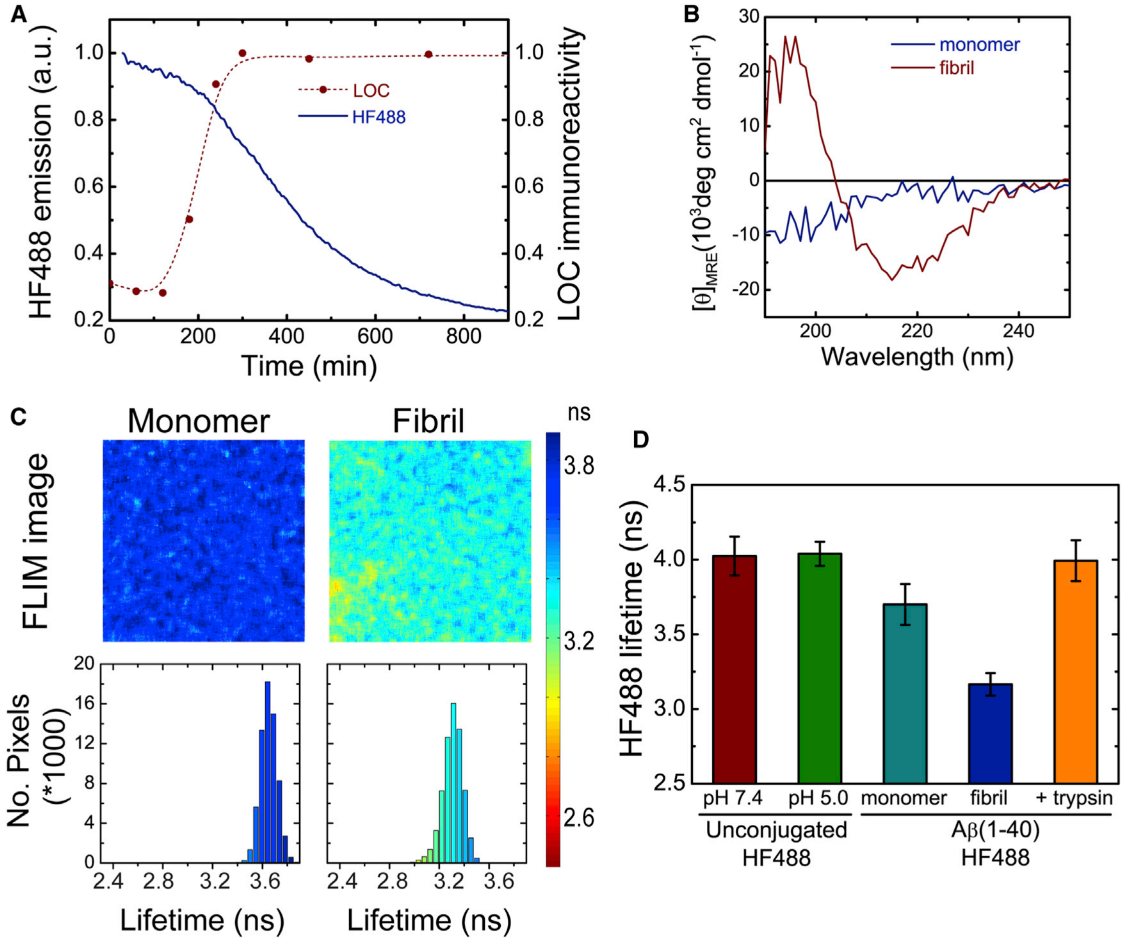

Figure 1. Amyloid Fibril Formation and Fluorescence Lifetimes of the HF488-Labeled Aβ Peptides In Vitro

(A) Amyloid formation by ^{HF488}Aβ(1–42) (5 μM in 50 mM sodium phosphate buffer [pH 7.4]) as a function of time. The quantity of amyloid fibrils was monitored by immunochemistry using the conformation-specific antibody LOC (red circles and dashed line) to detect fibrillar species. The normalized fibril quantity (relative to the maximum value) is given on the right axis in the graph. The amyloid formation was also monitored by the decrease in fluorescence emission intensity of the HF488 dye (blue). a.u., arbitrary units. See also Figure S1A.

(B) Far UV circular dichroism spectra of monomeric and fibrillar Aβ(1–42).

(C) Fluorescence lifetime images of solutions of monomeric and fibrillar ^{HF488}Aβ(1–42). The lifetime color coding is shown in the bar to the right of the images. The frequency histograms show the per-pixel distribution of HF488 fluorescence lifetimes in each of the images.

(D) Mean fluorescence lifetime (± SD) of the unconjugated HF488 dye at different pH values and for monomeric, fibrillar, and trypsin-treated monomeric ^{HF488}Aβ(1–40).

increase, the dye can be assumed to remain attached to the intact peptide chain. Taken together, therefore, these data suggest that the fluorescence lifetime of the HF488 dye constitutes a robust and specific reporter of the formation of amyloid fibrils by the Aβ peptides.

To further test the potential of the HF488 dye to report on the aggregation behavior of Aβ and the extent of amyloid formation, we examined the degree to which the changes in the mean fluorescence lifetime reflect the kinetics of amyloid formation in a sample of ^{HF488}Aβ(1–40) mounted on the stage of the FLIM microscope (Figure 2). The decrease in fluorescence lifetime is sigmoidal and proceeded by a lag phase of approximately 2.5 hr (Figure S2A). The observed kinetics are in good agreement with the appearance of LOC immunoreactive species (Figure S2B), although the change in fluorescence lifetime is slower than the increase in LOC species, a result consistent with the data presented in Figure 1. In addition, the fluorescence lifetime distribution broadens as the aggregation progresses, consistent with the appearance of an increasingly heterogeneous population of ^{HF488}Aβ(1–40) aggregates.

Uptake and Intracellular Localization of ^{HF488}Aβ(1–40) and ^{HF488}Aβ(1–42)

Aβ peptides have been reported to be internalized into a variety of cell types, including neurons (Burdick et al., 1997; Friedrich et al., 2010; Hu et al., 2009; Kaminski Schierle et al., 2011b; McGuire et al., 2012). To characterize the uptake and intracellular localization of the peptides under the conditions used in our study, we added aliquots of monomeric ^{HF488}Aβ(1–40) or ^{HF488}Aβ(1–42) to cultures of SH-SY5Y human neuroblastoma

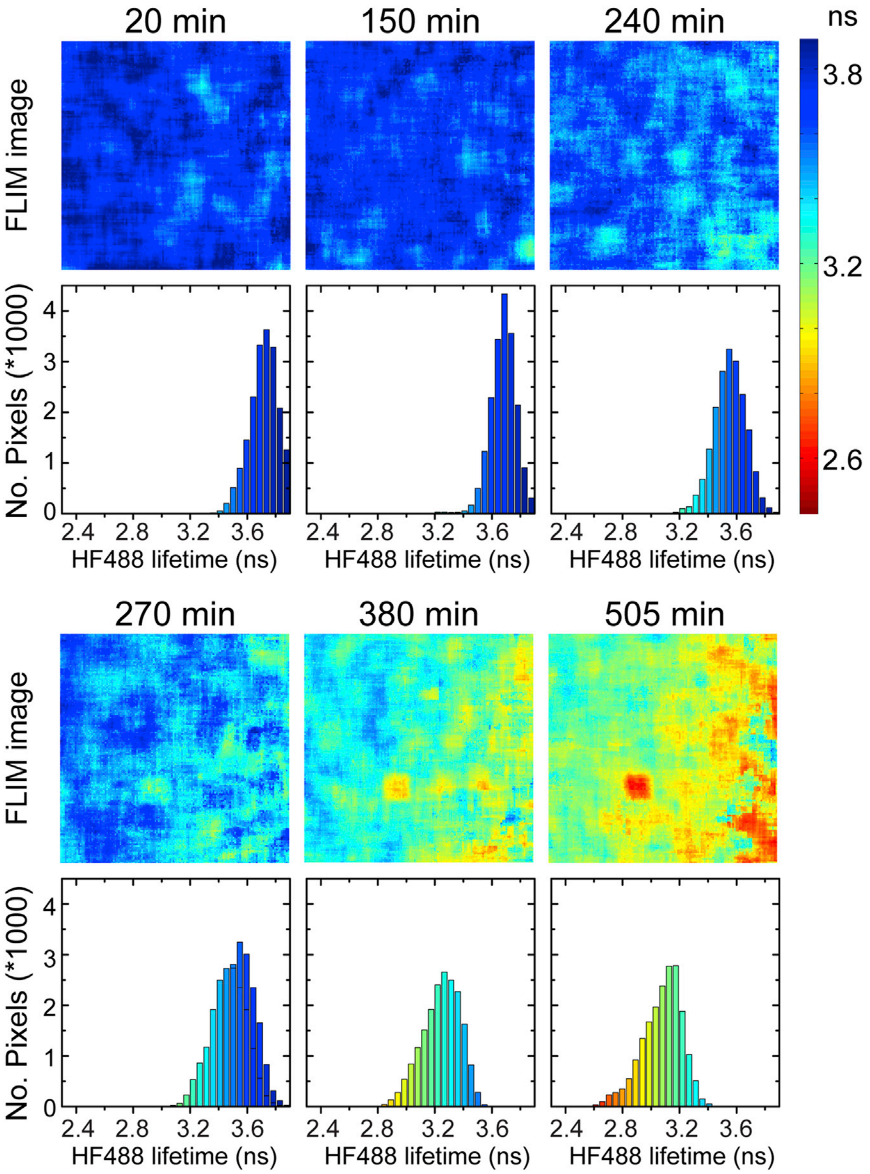

Figure 2. Kinetics of ^{HF488}Aβ(1–40) Fibril Formation Monitored by Fluorescence Lifetime Imaging
Fluorescence lifetime images and corresponding frequency histograms showing the evolution of the HF488 fluorescence lifetime in a defined volume of a droplet sample of ^{HF488}Aβ(1–40) deposited onto a glass-bottomed culture dish and mounted on the FLIM microscope. The color coding of images and histograms relate to the color bar on the right. The ^{HF488}Aβ(1–40) concentration was 5 μM, and the peptide was diluted into 50 mM sodium phosphate buffer (pH 7.4). See also Figure S2.

cells to give a total peptide concentration of 500 nM in the cellular medium. Intracellular green HF488 fluorescence, which was colocalized with the red fluorescence from the membrane-impermeable endocytosis marker FM 4–64, was observed readily after 30 min of exposure to the labeled peptides (Figures 3A and 3C).; Moreover, lower levels of intracellular HF488 fluorescence could be detected after 10 min (Figure S3), indicating rapid internalization of the peptides. The absence of enhanced HF488 fluorescence (green) in the plasma membrane (brightly stained in red by the hydrophobic FM 4–64 dye) compared with the fluorescence intensity in the surrounding extracellular medium suggests that neither ^{HF488}Aβ(1–40) nor ^{HF488}Aβ(1–42) accumulates significantly at the plasma membrane prior to internalization. Images acquired after 24 hr show that both ^{HF488}Aβ(1–40) and ^{HF488}Aβ(1–42) appear as granular intracellular deposits and that the peptides colocalize with LysoTracker red, confirming their compartmentalization within acidic organelles (Figures 3B and 3C). The neuronal cells were also incubated for 1 hr at 37°C and at 4°C to compare uptake under conditions that, respectively, permit or inhibit endocytosis. The cells did not internalize any detectible quantities of either ^{HF488}Aβ(1–40) or ^{HF488}Aβ(1–42) at 4°C, suggesting that uptake of the monomeric Aβ peptides occurs via an active uptake pathway (Figure 3E).

Intracellular Aggregation of Aβ(1–40) and Aβ(1–42) Monitored by Live Cell Fluorescence Lifetime Imaging

Having established that the fluorescence lifetime of HF488 acts as a reporter of amyloid formation of Aβ(1–40) and Aβ(1–42), we set out to investigate how the aggregation state of each of the two peptide isoforms is affected by its cellular uptake and accumulation in acidic organelles in SH-SY5Y cells. Using FLIM, we observed that there are no significant differences ($p = 0.95$, Table S1) in the fluorescence lifetimes of extracellular and internalized ^{HF488}Aβ(1–40) during the first 6 hr after application of 500 nM peptide to the culture medium (Figures 4A and 4D), indicating the presence of similar ^{HF488}Aβ(1–40) species within the cells as those applied to the extracellular environment. The fluorescence intensity images, however, show that the developing intracellular fluorescence becomes significantly brighter than the extracellular fluorescence, which clearly indicates that the ^{HF488}Aβ(1–40) peptide molecules have accumulated within the cells over this time period. We then examined the cells treated with the same concentration of ^{HF488}Aβ(1–40) for 24 and 48 hr (Figures 4C and 4D), washing them prior to imaging to remove any extracellular fluorescence. The bar diagram in Figure 4D shows that the fluorescence lifetime of the intracellular peptide decreases over this time interval. The difference compared with the extracellular starting value, however, is statistically significant first after 48 hr ($p = 0.0032$, Table S1).

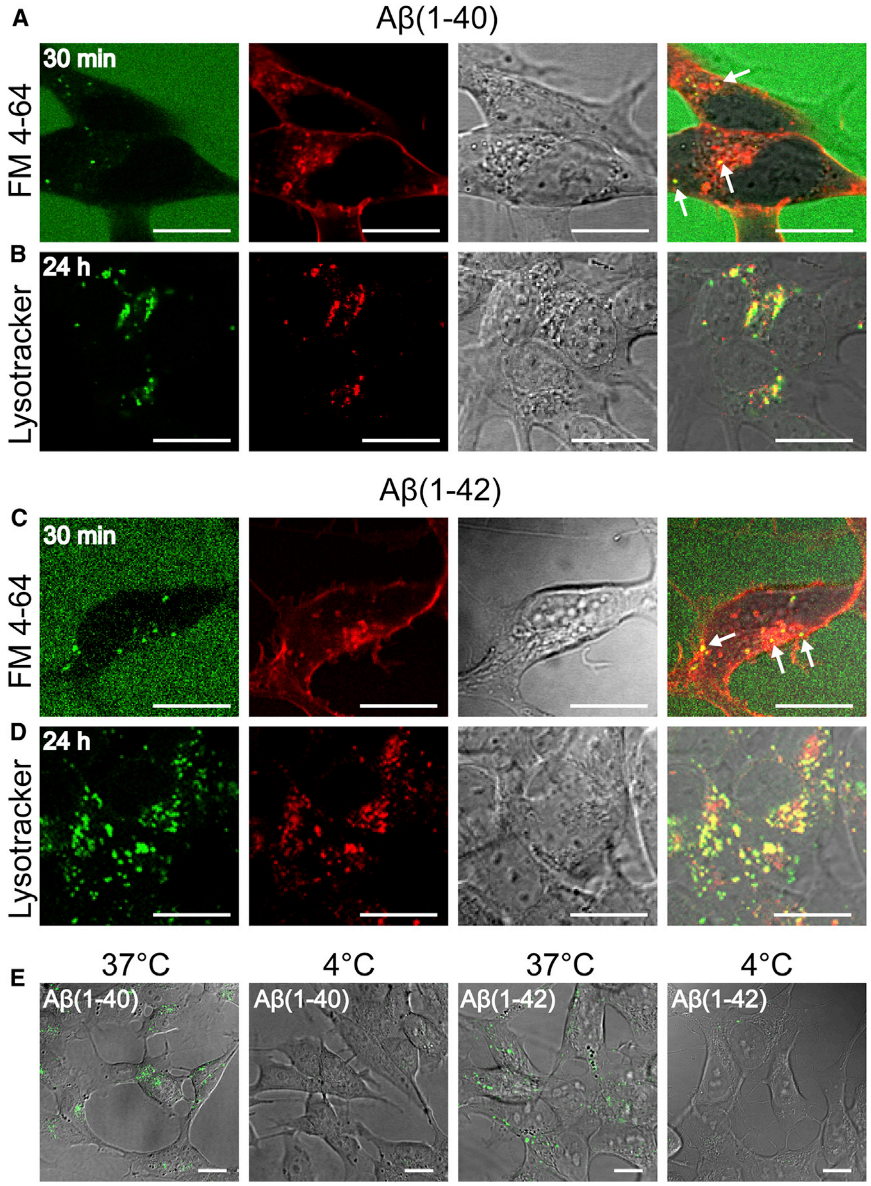

Figure 3. Confocal Fluorescence Microscopy Images Showing the Uptake and Intracellular Localization of ^{HF488}Aβ(1–40) and ^{HF488}Aβ(1–42) in SH-SY5Y Cells

(A and C) Uptake of (A) ^{HF488}Aβ(1–40) and (C) ^{HF488}Aβ(1–42) (green) and the endocytosis marker FM 4–64 (red) after 30 min of incubation. The white arrows in the overlay panels indicate colocalization in intracellular vesicles. Note that the peptides do not accumulate at the plasma membrane.

(B and D) Colocalization of (B) ^{HF488}Aβ(1–40) and (D) ^{HF488}Aβ(1–42) (green) with LysoTracker red (red) after 24 hr of incubation with 500 nM peptide followed by 1 hr of incubation with 100 nM LysoTracker red.

(E) Uptake of ^{HF488}Aβ(1–40) and ^{HF488}Aβ(1–42) upon incubation at 37°C (allowing endocytotic internalization) and 4°C (inhibiting endocytotic internalization). The images show the overlay of HF488 fluorescence (green) and the transmitted image. All scale bars represent 10 μm. See also Figure S3.

By contrast, after incubating cells with 500 nM ^{HF488}Aβ(1–42), we observed a trend of a progressive intracellular fluorescence lifetime decrease with time (Figures 4B and 4D). Indeed, the difference compared with the extracellular fluorescence lifetime at time 0 is statistically significant at the 6-hr time point ($p = 0.0028$, Table S1), and, after 24 hr, the lifetime has reached its lowest value (Figure 4C). There is, therefore, a clear difference between the two peptides with respect to the kinetics by which intracellular species with reduced fluorescence lifetimes are formed. ^{HF488}Aβ(1–42) displays a faster fluorescence lifetime decay than ^{HF488}Aβ(1–40), although both reach endpoint values (3.35 ± 0.2 ns for ^{HF488}Aβ(1–40) compared with 3.31 ± 0.2 ns for ^{HF488}Aβ(1–40) that are statistically indistinguishable ($p = 0.22$, Table S2). In addition, ^{HF488}Aβ(1–40) appears to exhibit a lag phase in its aggregation, whereas ^{HF488}Aβ(1–42) shows no such effect and aggregates rapidly soon after it is internalized into the cells (Figure 4D).

Direct Stochastic Optical Reconstruction Microscopy of ^{HF647}Aβ(1–40) and ^{HF647}Aβ(1–42) in Cells

To further investigate the nature of the intracellular Aβ(1–40) and Aβ(1–42) aggregates, we used superresolution fluorescence microscopy to analyze their physical appearance. We have demonstrated recently that it is possible to obtain single-molecule localization of Hilyte Fluor 647 fluorophores attached to preassembled or spontaneously formed amyloid fibrils in fixed cells using direct stochastic optical reconstruction microscopy (*d*STORM) (Kaminski Schierle et al., 2011b). Thus, we treated SH-SY5Y cells with 500 nM of ^{HF647}Aβ(1–40) or 500 nM of ^{HF647}Aβ(1–42) monomers for periods of 24 and 48 h. Thereafter, the cells were fixed, immersed in switching buffer, and imaged at single-molecule resolution using a homebuilt total internal reflection fluorescence (TIRF) microscope operated under highly inclined illumination (Tokunaga et al., 2008) to obtain images from an image plane within the cells. Superresolved images (Figure 5A) of the intracellular ^{HF647}Aβ(1–40) and ^{HF647}Aβ(1–42) aggregates were then reconstructed as described in the Experimental Procedures. *d*STORM results in a dramatic increase in resolution compared with conventional confocal imaging (Figure S4). In this case, the lateral resolution of our images was calculated to be 55–70 nm using a blind assessment density estimation approach (Rees et al., 2012). Features larger than the lateral resolution limit can, therefore, be interpreted with confidence as having approximately the correct size and shape. The images taken after 24 hr (Figure 5A) show that the intracellular ^{HF647}Aβ(1–40) aggregates are mainly spherical, and we estimated their average

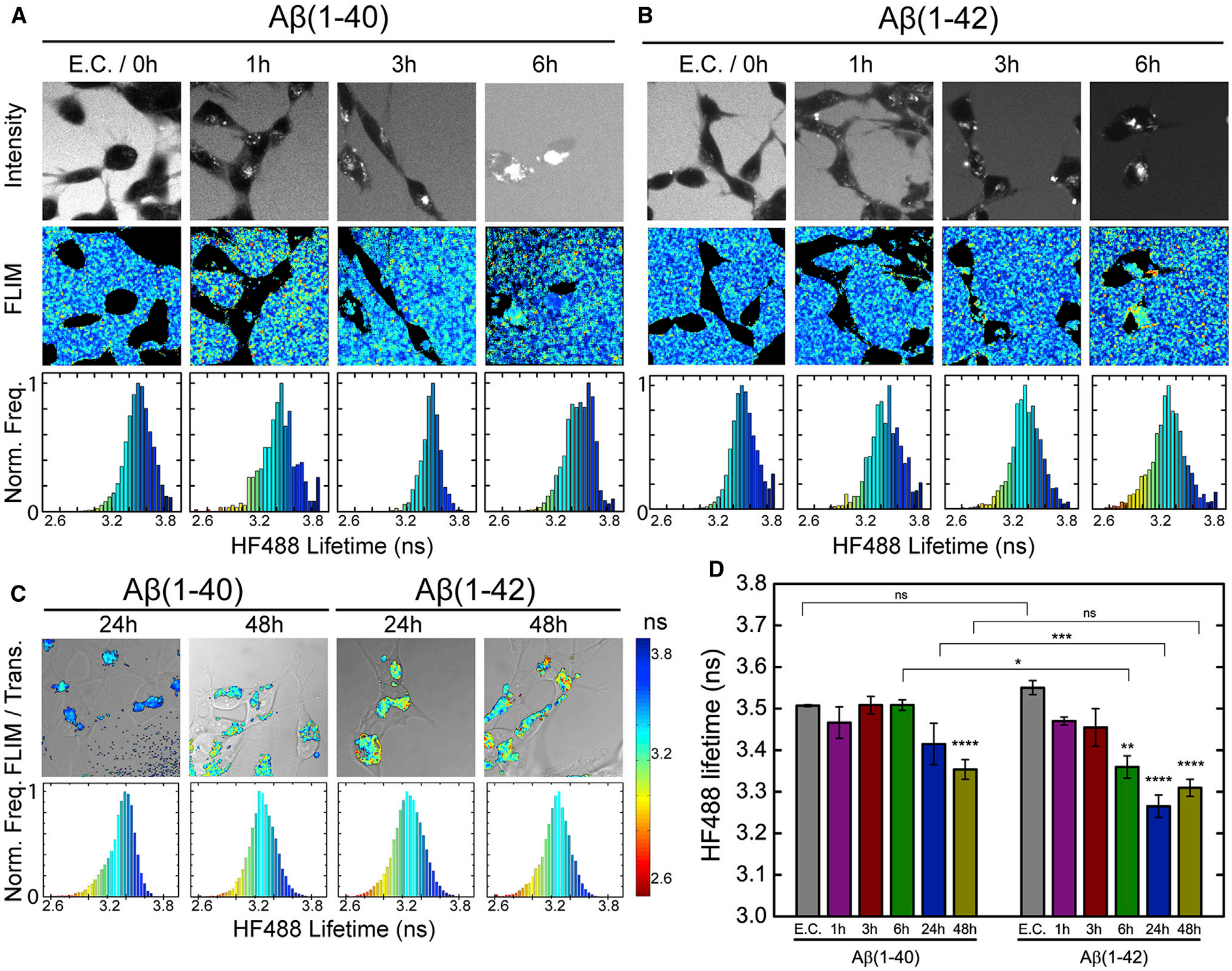

Figure 4. Fluorescence Lifetime Imaging of the Uptake and Accumulation of ^{HF488}Aβ(1–40) and ^{HF488}Aβ(1–42) in Live SH-SY5Y Cells

(A and B) Cells treated with (A) 500 nM ^{HF488}Aβ(1–40) or (B) 500 nM ^{HF488}Aβ(1–42) for 0–6 hr. The upper panels show fluorescence intensity, the center panels show HF488 fluorescence lifetimes, and the lower panels show the normalized per-pixel HF488 fluorescence lifetime frequency (Norm. Freq.) in the corresponding image above. The histograms at the 0 hr time point show the HF488 fluorescence lifetime distribution of the extracellular peptides (E.C.). Subsequent histograms (1, 3, and 6 h) show the HF488 fluorescence lifetime distributions of the intracellular peptides only. See the color bar in (C) for the color coding of FLIM images and frequency histograms.

(C) Cells treated with 500 nM ^{HF488}Aβ(1–40) or 500 nM ^{HF488}Aβ(1–42) for 24 or 48 hr. The cells were washed once prior to imaging. The images show an overlay of the color-coded FLIM image and the transmitted image. The HF488 fluorescence lifetime frequency histograms show the distribution over several analyzed images (n = 6–9). Trans., transmission.

(D) Mean HF488 fluorescence lifetime (± SEM) of extracellular and intracellular ^{HF488}Aβ(1–40) and ^{HF488}Aβ(1–42). At 0 hr, the bars represent the mean HF488 fluorescence lifetime (± SEM) of the extracellular peptide (E.C.). The subsequent bars show the mean HF488 fluorescence lifetime (± SEM) of the intracellular peptides at the indicated time points after addition of 500 nM ^{HF488}Aβ(1–40) or 500 nM ^{HF488}Aβ(1–42) to the culture medium. Asterisks (*) over bars indicate a significant difference compared to 0 h (E.C.), and lines over bars indicate a significant difference between the two peptides at the specified time points according to Fisher's least significant difference test ($p < 0.05$). *$p < 0.05$; **$p < 0.01$; ***$p < 0.001$; ****$p < 0.0001$. Individual p values are given in Tables S1 and S2.

diameter to be approximately 160 nm (Figure 5B) using pixel counting. The ^{HF647}Aβ(1–42) aggregates, by contrast, appear to include both spherical and elongated species, with an average size along their longest dimension of approximately 225 nm (Figure 5B). The observation that, after 24 hr, ^{HF647}Aβ(1–42) forms intracellular species that are larger than those formed by ^{HF647}Aβ(1–40) is consistent with the differences in their mean fluorescence lifetimes (Figures 4C, 4D, and 5C). Together, these observations suggest a higher degree of conversion into amyloid fibrils after 24 hr for ^{HF647}Aβ(1–42) in relation to ^{HF647}Aβ(1–40). After 48 hr, the ^{HF647}Aβ(1–40) aggregates remain predominantly spherical, but they appear to have increased in size (Figure 5B), suggesting a continued recruitment of monomers into the amyloid aggregates, which is consistent with the fluorescence lifetime change observed for ^{HF488}Aβ(1–40) between 24 and 48 hr (Figures 4C, 4D, and 5C). There is no significant change in the

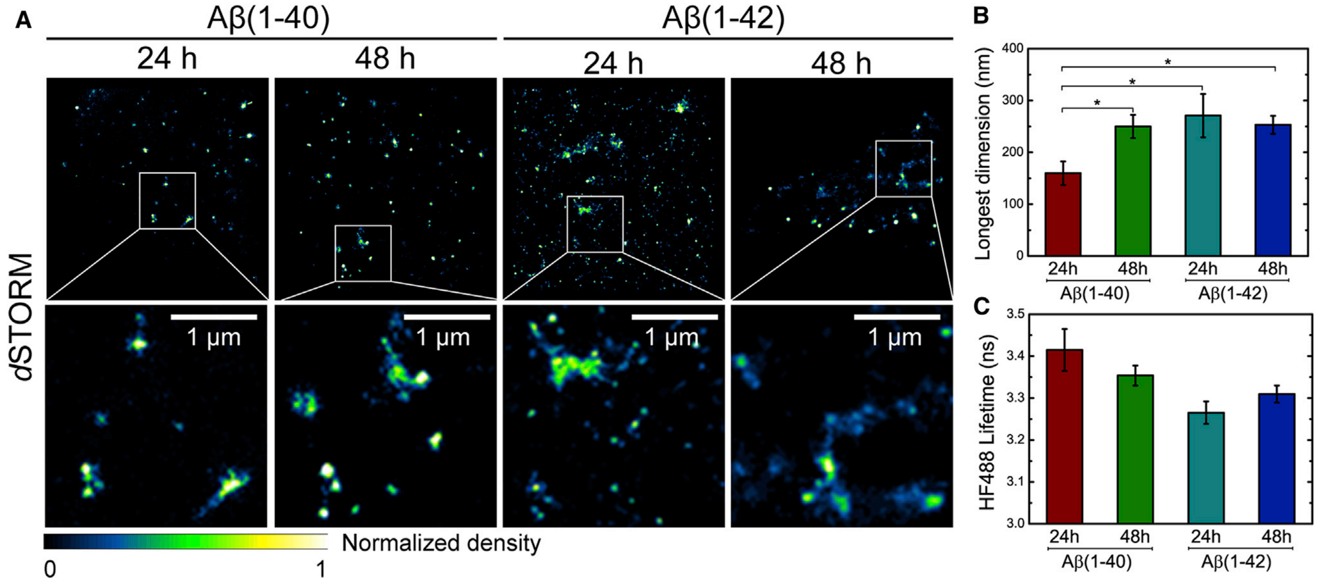

Figure 5. Super-Resolution Fluorescence Microscopy Imaging of Intracellular Aβ(1–40) and Aβ(1–42)

(A) *d*STORM images of intracellular HF647Aβ(1–40) and HF647Aβ(1–42) in fixed SH-SY5Y cells. The cells were imaged after 24 and 48 hr of incubation. Scale bars represent 1 μm.

(B) Mean size of the intracellular aggregates (± SEM) estimated by pixel counting in *d*STORM images. The lines above the bars indicate statistically significant mean differences (paired Student's t test; *$p < 0.05$; $n = 18$–27). Only aggregates with dimensions exceeding the resolution limit were included in the analysis.

(C) Mean fluorescence lifetime (± SEM) of intracellular HF488Aβ(1–40) and HF488Aβ(1–42) at the time points corresponding to the *d*STORM images. The data were extracted from Figure 4D.

size of HF647Aβ(1–42) aggregates between 24 and 48 hr, again indicating a correlation consistent with the FLIM results. Comparison of the FLIM and *d*STORM data recorded after 24 and 48 hr indicates a qualitative relationship between aggregate size and HF488 fluorescence lifetime (Figures 5B and 5C), supporting the conclusion that the fluorescence lifetime reflects the nature and abundance of the Aβ amyloid aggregates formed in vivo.

DISCUSSION

The misfolding and formation of amyloid aggregates by Aβ peptides, particularly Aβ(1–42), is now well established as a central and causative step in the onset and progression of the molecular transformations leading to neurodegeneration and, ultimately, to the development of AD. The ability to directly monitor by experiment the dynamics of the aggregation process in live specimens is, therefore, a crucial step in defining the nature of the molecular events that initiate the assembly and proliferation of Aβ aggregates in vivo. We describe here a combination of fluorescence-based microscopy techniques designed to overcome the challenging nature of such experiments that have enabled us to study, in real time, the dynamics by which fluorescently labeled Aβ peptides form aggregates within acidic organelles of the endocytotic pathway of live neurons and to characterize their amyloidogenic nature and size.

In this study, we confirm results from others indicating that both Aβ(1–40) and Aβ(1–42) are taken up and then accumulate in human neuronal cells (Hu et al., 2009). We show, in addition, that this uptake is rapid and spontaneous, that it occurs without any apparent accumulation of peptide molecules in the plasma membrane, that it is temperature-dependent, and that the internalized peptide molecules colocalize with markers for endocytosis and acidic vesicular organelles. Taken together, these observations indicate that monomeric Aβ(1–40) and Aβ(1–42) are both taken up into the cells as monomers and via active endocytotic pathways, although it has been shown that other amyloid species, including oligomers (Campioni et al., 2010) and fibrils (Kaminski Schierle et al., 2011b; McGuire et al., 2012), if present in the cellular medium, can also be taken up via nonendocytotic pathways. We observe retention of Aβ in acidic compartments over 48 hr, suggesting their localization in lysosomes in accord with the findings of several previous studies (Burdick et al., 1997; Hu et al., 2009; Knauer et al., 1992). By monitoring the changes in fluorescence lifetimes of internalized HF488Aβ(1–40) and HF488Aβ(1–42), we have been able to show that the accumulation of monomeric species into acidic organelles results in the gradual formation of Aβ aggregates, as demonstrated by the decrease in fluorescence lifetime of intracellular Aβ as a function of time.

Despite the apparent similarities in observed endocytotic uptake and intracellular accumulation of HF488Aβ(1–40) and HF488Aβ(1–42), our findings reveal substantial differences in the kinetic rate profiles by which the two Aβ isoforms form intracellular aggregates within the vesicular compartments of the cell. In short, intraneuronal HF488Aβ(1–40) does not exhibit detectable changes in its fluorescence lifetime during the first 6 hr of incubation, although a decrease in fluorescence lifetime is evident after 48 hr. This rate profile indicates the existence of a significant lag phase for intracellular Aβ(1–40) aggregation. By contrast,

intraneuronal ^{HF488}Aβ(1–42) exhibits a progressive decrease in mean fluorescence lifetime, despite showing a distinctive, although short, lag phase in vitro (Figure 1), in agreement with previous studies (Cohen et al., 2013). Thus, although the overall slower aggregation kinetics of intracellular ^{HF488}Aβ(1–40) relative to intracellular ^{HF488}Aβ(1–42) are consistent with their intrinsic aggregation propensities (Jarrett et al., 1993), our results reveal an important difference in the details of their aggregation reactions in vivo, where, in particular, ^{HF488}Aβ(1–42) appears to proceed to form amyloid fibrils without a lag phase.

The absence of a detectable lag phase in the case of ^{HF488}Aβ(1–42) cannot be interpreted definitely from this experiment, although it is likely to be associated with the fact that the aggregation proceeds in lysosomes, where the environment is relatively acidic. This pH change brings the peptide closer to its isoelectric point, and several in vitro studies indeed suggest that this results in strongly accelerated Aβ amyloid formation (Burdick et al., 1992; Su and Chang, 2001; Wood et al., 1996). A more detailed interpretation of the rate profiles would require detailed knowledge of the intracellular concentration and of the mechanism of nucleation that exist in the cellular environments (Cohen et al., 2013; Cohen et al., 2012). Nevertheless, our findings are consistent with the observation that neurons in regions of the brain that are most vulnerable in AD accumulate Aβ(1–42) preferentially relative to Aβ(1–40), although the latter peptide isoform is more abundant (Gouras et al., 2000). The demonstration of the rapid formation of Aβ(1–42) aggregates in vivo is also particularly interesting in light of a recent in vitro study that has revealed that the presence of such aggregates can result in the rapid generation of toxic oligomeric species through catalytic secondary nucleation (Cohen et al., 2013).

In addition to studying the kinetics of amyloid formation by FLIM in vivo, we also used superresolution fluorescence imaging to examine the size and shape of the resulting Aβ aggregates in situ. We can conclude that the uptake of monomeric Aβ and its subsequent amyloid formation result in the formation of relatively small and compact amyloid structures. Their physical size is of the order of 150–300 nm along their longest dimension (Figure 5B), and their macroscopic structures are, in most cases, too dense to discern individual fibrils (although we can observe some higher aspect ratio structures for ^{HF647}Aβ(1–42)). These observations contrast with the extended shape of well separated fibrils formed by these Hilyte-labeled peptides in vitro (Narayan et al., 2012) and suggests that spatial confinement within cellular compartments has a large influence on the macroscopic structure of the assembled fibrils. In this context, however, it is interesting to compare these findings to our previous *d*STORM studies of intracellular Aβ, in which we incubated HeLa cells with a mixture of monomers and short, preformed fibrils (Kaminski Schierle et al., 2011b) and, thereafter, observed rapid fibril growth and clearly discernable individual fibrils within cells.

Finally, we note a qualitative correlation between the average sizes of intracellular ^{HF647}Aβ aggregates and the mean fluorescence lifetimes of the corresponding ^{HF488}Aβ aggregates. This observation supports the view that fluorescence lifetime is a sensitive measure of the extent of amyloid formation both in vitro and in vivo.

SIGNIFICANCE

We demonstrated that it is possible to measure, noninvasively, the kinetics of the process by which fluorescently labeled Aβ(1–40) and Aβ(1–42) peptides form amyloid aggregates in acidic vesicular compartments within live neurons following the endocytotic uptake of Aβ monomers from the extracellular medium. By this approach, we identified differences in the self-assembly of intraneuronal Aβ(1–40) and Aβ(1–42), with the latter aggregating more rapidly than the former and without any detectable lag phase. We also imaged the macroscopic structure of the ensuing Aβ aggregates in situ and showed that their physical size correlates qualitatively with the extent to which each peptide has adopted an amyloid structure. This work demonstrates that it is possible to resolve the structural dynamics of the assembly of Aβ aggregates in vivo, and we envisage that this approach will significantly enhance our ability to define and understand the mechanisms through which this key event in the development of AD can occur. We envisage that the latter will increase the likelihood of finding strategies to design rational approaches for therapeutic interventions directed at combatting the onset and progression of neurodegenerative conditions.

EXPERIMENTAL PROCEDURES

Reagents

Synthetic Aβ(1–40) and Aβ(1–42) peptides, labeled at the N terminus with HF488 or HF647 and Hilyte Fluor 488 acid (free dye) were purchased from Anaspec. The fibril-specific LOC antibody (rabbit polyclonal, catalog no. AB2287) was obtained from Millipore, and the secondary Alexa Fluor 647-labeled anti-rabbit antibody was obtained from Life Technologies. The human neuroblastoma SH-SY5Y cells were from Sigma.

Preparation and Handling of the Aβ Peptides

The lyophilized peptides were dissolved in 1% ammonium hydroxide (v/v) at 4°C. The solutions were then vortexed briefly and divided into aliquots that were immediately frozen and stored below −20°C until further use to avoid any aggregation. The concentration of the peptides in the aliquots was determined by absorption spectroscopy using an extinction coefficient of 70,000 $M^{-1}cm^{-1}$ at 503 nm for the HF488 dye and an extinction coefficient of 250,000 $M^{-1}cm^{-1}$ at 649 nm for the HF647 dye. A fresh aliquot was used for each experiment to avoid repeated freeze-thaw cycles that could trigger potential aggregate formation.

Aβ Peptide Aggregation in Vitro

All in vitro experiments were performed at a peptide concentration of 5 μM in 50 mM sodium phosphate buffer at pH 7.4. Aggregation kinetics were monitored by steady-state fluorescence in a FLUOstar Optima microplate reader (BMG Labtech) and sealed black Costar half-area, 96-well plates (Corning Life Sciences). Data were recorded at 5-min intervals using a 480-nm bandpass filter for excitation and a 520-nm bandpass filter for emission. Orbital shaking (300 rpm, 10 s) was carried out prior to each recording to ensure that each sample was homogenous. Sample aliquots for dot blot analysis were taken at the indicated time points and immediately flash-frozen in liquid nitrogen. These aliquots were thawed by dilution into phosphate buffer to a total volume of 25 μl and immediately blotted onto a nitrocellulose membrane using a Bio-Rad dot blot apparatus. The membrane was blocked with 5% (w/v) dry milk and 0.1% Triton X-100 in PBS and stained with LOC as the primary antibody and an Alexa Fluor 647-labeled anti-rabbit antibody as the secondary antibody (Life Technologies). The blots were imaged on a Typhoon 9400 scanner (GE Healthcare) using the 633-nm laser line for excitation. Densitometric analysis of the blots was carried out using ImageJ. (Schneider et al., 2012).

Aβ samples for in vitro fluorescence lifetime imaging were placed in a glass-bottomed tissue culture dish (MatTek) and sealed with a coverslip and nail varnish to avoid evaporation.

Optical Spectroscopy

Absorption spectra were recorded on a Cary 400 UV-Vis spectrophotometer (Agilent). Circular dichroism spectra were recorded on a J-810 spectropolarimeter (JASCO) between 190–250 nm in 1-nm wavelength increments with a scan speed of 50 nm/min, a response time of 0.5 s, and a bandwidth of 2 nm. Twenty scans were accumulated and averaged. Spectra were corrected for background contributions by subtracting the appropriate blanks.

Cell Culture and Sample Preparation

Human neuroblastoma cells (SH-SY5Y) were grown in 1:1 minimal essential medium (MEM) and nutrient mixture F-12 Ham (Sigma-Aldrich) with sodium bicarbonate, including 15% heat-inactivated fetal bovine serum, 1% MEM non-essential amino acids, 2 mM N-glutamine, 1% penicillin-streptomycin (10,000 U/ml K1), and 0.1% fungizone (amphotericin B, 250 mg/ml K1) (Life Technologies). Cells were plated 1 day prior to experiments in glass-bottomed culture dishes (MatTek) at a density of 50,000 cells/14-mm dish. All samples were washed once in CO_2-independent, serum- and phenol red-free medium (prepared as above but with exclusion of serum and addition of 30 mM HEPES and B12 supplement [Gibco]). All experiments with cells were performed in at least duplicates and repeated at least three times. The Aβ concentration was 500 nM in all experiments.

Confocal Microscopy

All uptake and colocalization experiments were carried out with the HF488-labeled Aβ peptides using a Leica SP5 confocal microscope (Leica Microsystems). For colocalization with FM 4–64 (Life Technologies), 5 μg/ml of the dye was added together with the peptide, and images were recorded after 1 hr of incubation. Both dyes were excited by the 488-nm laser line. HF488 emission was collected from 520–580 nm and FM 4–64 emission between 700–800 nm. For colocalization with LysoTracker red (Life Technologies), cells were incubated with HF488-labeled peptide for 24 hr, washed once with serum-free medium, and thereafter incubated for 1 hr with 100 nM LysoTracker red before images were recorded. LysoTracker red was excited using the 647-nm laser line, and emission was collected between 700–800 nm. Sequential acquisition was used in all experiment to avoid bleed-through and cross-excitation. Cellular uptake at 4°C was assessed by keeping the cells in a cold room during incubation. The cells were washed prior to imaging to avoid uptake while observing the cells on the microscope.

FLIM

All FLIM experiments were carried out using HF488-labeled Aβ peptides, and images were recorded by time-correlated single photon counting (TCSPC) using a confocal microscopy platform built on an Olympus Fluoview FV300 confocal scan unit. A pulsed supercontinuum source (SC 450, Fianium) was used for excitation, emitting a train of sub-10-picosecond pulses at 40 MHz repetition rate. The output beam was collimated and passed through a hot mirror assembly to remove infrared components at wavelengths greater than 700 nm. The visible portion of the spectrum was passed through an acousto-optic tunable filter (AOTFnC-VIS, AA Opto-Electronic) whose radio frequency modulator was driven by software developed using LabVIEW (National Instruments). The excitation wavelength was set to 480 nm. The excitation beam was reflected onto the sample with a 20/80 broad bandwidth coated beam splitter so that 20% of the excitation light passed on to the sample and 80% of the fluorescence signal was reflected toward the confocal pinhole. The fluorescence light was passed through a 515-nm Semrock long-pass filter (Laser 2000) and passed onto a fast photomultiplier tube (PMC-100, Becker & Hickl). Lifetimes were recorded using TCSPC circuitry (SPC-830, Becker & Hickl). Photon count rates were kept below 1% of the laser repetition rate to prevent pulse pileup. Images were acquired in 10 cycles of 10–30 s. All TCSPC images were processed initially using SPCImage software (Becker & Hickl) and fitted as monoexponential decays, taking into account the instrument response. The instrument response was measured using reflected light from either a coverslip or a coated mirror. Pixel binning was increased until approximately 3500–5000 photons/pixel were obtained (corresponding to a binning factor of 2 or 3). Further image analysis was carried out using Matlab (The Mathworks) and Origin 8.1 (OriginLab) software. Statistical analysis was performed in Origin 8.1 (OriginLab) or GraphPad Prism (GraphPad Software). The average lifetimes in several independent images were computed, and these were then averaged to obtain the mean lifetime (± SEM) for each particular time point and peptide treatment. Significant differences between treatments were obtained by means of comparison using paired Student's t test or Fisher's least significant difference test (in case of multiple comparisons). The p values for all individual comparisons are given in Tables S1 and S2.

*d*STORM

All *d*STORM experiments were carried out using HF647-labeled peptides. Fluorescence images for *d*STORM were taken on a Nikon Te-300 Eclipse inverted wide field microscope, with a 100×, 1.49 numerical aperture TIRF objective lens, an Andor iXon 887DV electron-multiplying charge-coupled device camera, and a Semrock fluorescence filter set (LF405/488/561/635-A-00). To induce photoswitching, the specimens were immersed in a "switching buffer" solution: 100 mM mercaptoethylamine in PBS (pH 7.4) together with a glucose enzyme oxygen scavenger (40 mg/ml glucose, 50 μg/ml glucose oxidase, and 1 μg/ml catalase) to slow down photobleaching of the fluorophore. Samples were imaged using highly inclined illumination from a 642-nm Toptica diode laser at an intensity of ~2 kW/cm^2 on the specimen. Stacks of 10,000 images with 25 ms exposure times were collected.

Superresolution images were reconstructed by applying a "segmentation and sparse Gaussian fitting" algorithm implemented in Matlab using a method described previously (Wolter et al., 2010). Briefly, for each frame of image data, local maxima brighter than a threshold value were fitted to a Gaussian point spread function to obtain the center position corresponding to a fluorophore location. Localized fluorophore density was visualized by assigning each localization onto a superresolution grid of pixels to produce a fluorophore density histogram as the superresolution image. The number of photons associated with each localization and the background noise of the images were also estimated so that the average localization precision could be evaluated according to Thompson et al. (Thompson et al., 2002) and used to determine the image resolution (Rees et al., 2012). The sizes of intracellular Aβ aggregates were estimated from the *d*STORM images by counting pixels along their longest dimension. Only aggregates with dimensions exceeding the resolution limit of the experiment were included in the analysis.

SUPPLEMENTAL INFORMATION

Supplemental Information includes four figures and two tables and can be found with this article online at http://dx.doi.org/10.1016/j.chembiol.2014.03.014.

ACKNOWLEDGMENTS

E.K.E. holds a long-term postdoctoral and career development fellowship from the Wenner-Gren Foundation. C.F.K. acknowledges funding for this work from the EPSRC, the Alzheimer Research UK Trust, the Wellcome Trust, and the Medical Research Council. C.M.D. acknowledges funding from the Wellcome Trust and the Medical Research Council. We thank Rebecca Barber for maintaining the cell cultures used in these studies.

Received: November 6, 2013
Revised: March 12, 2014
Accepted: March 24, 2014
Published: May 22, 2014

REFERENCES

Billings, L.M., Oddo, S., Green, K.N., McGaugh, J.L., and LaFerla, F.M. (2005). Intraneuronal Aβ causes the onset of early Alzheimer's disease-related cognitive deficits in transgenic mice. Neuron *45*, 675–688.

Burdick, D., Soreghan, B., Kwon, M., Kosmoski, J., Knauer, M., Henschen, A., Yates, J., Cotman, C., and Glabe, C. (1992). Assembly and aggregation properties of synthetic Alzheimer's A4/β amyloid peptide analogs. J. Biol. Chem. *267*, 546–554.

Burdick, D., Kosmoski, J., Knauer, M.F., and Glabe, C.G. (1997). Preferential adsorption, internalization and resistance to degradation of the major isoform of the Alzheimer's amyloid peptide, A β 1-42, in differentiated PC12 cells. Brain Res. *746*, 275–284.

Campioni, S., Mannini, B., Zampagni, M., Pensalfini, A., Parrini, C., Evangelisti, E., Relini, A., Stefani, M., Dobson, C.M., Cecchi, C., and Chiti, F. (2010). A causative link between the structure of aberrant protein oligomers and their toxicity. Nat. Chem. Biol. *6*, 140–147.

Capetillo-Zarate, E., Gracia, L., Yu, F., Banfelder, J.R., Lin, M.T., Tampellini, D., and Gouras, G.K. (2011). High-resolution 3D reconstruction reveals intra-synaptic amyloid fibrils. Am. J. Pathol. *179*, 2551–2558.

Chan, F.T., Kaminski Schierle, G.S., Kumita, J.R., Bertoncini, C.W., Dobson, C.M., and Kaminski, C.F. (2013). Protein amyloids develop an intrinsic fluorescence signature during aggregation. Analyst (Lond.) *138*, 2156–2162.

Chen, H., Ahsan, S.S., Santiago-Berrios, M.B., Abruña, H.D., and Webb, W.W. (2010). Mechanisms of quenching of Alexa fluorophores by natural amino acids. J. Am. Chem. Soc. *132*, 7244–7245.

Chiti, F., and Dobson, C.M. (2006). Protein misfolding, functional amyloid, and human disease. Annu. Rev. Biochem. *75*, 333–366.

Clifford, P.M., Zarrabi, S., Siu, G., Kinsler, K.J., Kosciuk, M.C., Venkataraman, V., D'Andrea, M.R., Dinsmore, S., and Nagele, R.G. (2007). Aβ peptides can enter the brain through a defective blood-brain barrier and bind selectively to neurons. Brain Res. *1142*, 223–236.

Cohen, S.I., Vendruscolo, M., Dobson, C.M., and Knowles, T.P. (2012). From macroscopic measurements to microscopic mechanisms of protein aggregation. J. Mol. Biol. *421*, 160–171.

Cohen, S.I., Linse, S., Luheshi, L.M., Hellstrand, E., White, D.A., Rajah, L., Otzen, D.E., Vendruscolo, M., Dobson, C.M., and Knowles, T.P. (2013). Proliferation of amyloid-β42 aggregates occurs through a secondary nucleation mechanism. Proc. Natl. Acad. Sci. USA *110*, 9758–9763.

Friedrich, R.P., Tepper, K., Rönicke, R., Soom, M., Westermann, M., Reymann, K., Kaether, C., and Fändrich, M. (2010). Mechanism of amyloid plaque formation suggests an intracellular basis of Aβ pathogenicity. Proc. Natl. Acad. Sci. USA *107*, 1942–1947.

Ghukasyan, V., Hsu, C.C., Liu, C.R., Kao, F.J., and Cheng, T.H. (2010). Fluorescence lifetime dynamics of enhanced green fluorescent protein in protein aggregates with expanded polyglutamine. J. Biomed. Opt. *15*, 016008.

Glenner, G.G., and Wong, C.W. (1984). Alzheimer's disease: initial report of the purification and characterization of a novel cerebrovascular amyloid protein. Biochem. Biophys. Res. Commun. *120*, 885–890.

Gouras, G.K., Tsai, J., Näslund, J., Vincent, B., Edgar, M., Checler, F., Greenfield, J.P., Haroutunian, V., Buxbaum, J.D., Xu, H., et al. (2000). Intraneuronal Aβ42 accumulation in human brain. Am. J. Pathol. *156*, 15–20.

Hardy, J., and Allsop, D. (1991). Amyloid deposition as the central event in the aetiology of Alzheimer's disease. Trends Pharmacol. Sci. *12*, 383–388.

Hu, X., Crick, S.L., Bu, G., Frieden, C., Pappu, R.V., and Lee, J.-M. (2009). Amyloid seeds formed by cellular uptake, concentration, and aggregation of the amyloid-β peptide. Proc. Natl. Acad. Sci. USA *106*, 20324–20329.

Jarrett, J.T., Berger, E.P., and Lansbury, P.T., Jr. (1993). The carboxy terminus of the β amyloid protein is critical for the seeding of amyloid formation: implications for the pathogenesis of Alzheimer's disease. Biochemistry *32*, 4693–4697.

Kaganovich, D., Kopito, R., and Frydman, J. (2008). Misfolded proteins partition between two distinct quality control compartments. Nature *454*, 1088–1095.

Kaminski Schierle, G.S., Bertoncini, C.W., Chan, F.T., van der Goot, A.T., Schwedler, S., Skepper, J., Schlachter, S., van Ham, T., Esposito, A., Kumita, J.R., et al. (2011a). A FRET sensor for non-invasive imaging of amyloid formation in vivo. ChemPhysChem *12*, 673–680.

Kaminski Schierle, G.S., van de Linde, S., Erdelyi, M., Esbjörner, E.K., Klein, T., Rees, E., Bertoncini, C.W., Dobson, C.M., Sauer, M., and Kaminski, C.F. (2011b). In situ measurements of the formation and morphology of intracellular β-amyloid fibrils by super-resolution fluorescence imaging. J. Am. Chem. Soc. *133*, 12902–12905.

Kayed, R., Head, E., Sarsoza, F., Saing, T., Cotman, C.W., Necula, M., Margol, L., Wu, J., Breydo, L., Thompson, J.L., et al. (2007). Fibril specific, conformation dependent antibodies recognize a generic epitope common to amyloid fibrils and fibrillar oligomers that is absent in prefibrillar oligomers. Mol. Neurodegener. *2*, 18.

Klucken, J., Outeiro, T.F., Nguyen, P., McLean, P.J., and Hyman, B.T. (2006). Detection of novel intracellular α-synuclein oligomeric species by fluorescence lifetime imaging. FASEB J. *20*, 2050–2057.

Knauer, M.F., Soreghan, B., Burdick, D., Kosmoski, J., and Glabe, C.G. (1992). Intracellular accumulation and resistance to degradation of the Alzheimer amyloid A4/β protein. Proc. Natl. Acad. Sci. USA *89*, 7437–7441.

Lee, J., Culyba, E.K., Powers, E.T., and Kelly, J.W. (2011). Amyloid-β forms fibrils by nucleated conformational conversion of oligomers. Nat. Chem. Biol. *7*, 602–609.

Lomakin, A., Chung, D.S., Benedek, G.B., Kirschner, D.A., and Teplow, D.B. (1996). On the nucleation and growth of amyloid β-protein fibrils: detection of nuclei and quantitation of rate constants. Proc. Natl. Acad. Sci. USA *93*, 1125–1129.

Masters, C.L., Simms, G., Weinman, N.A., Multhaup, G., McDonald, B.L., and Beyreuther, K. (1985). Amyloid plaque core protein in Alzheimer disease and Down syndrome. Proc. Natl. Acad. Sci. USA *82*, 4245–4249.

McGuire, E.K., Motskin, M., Bolognesi, B., Bergin, S.D., Knowles, T.P., Skepper, J., Luheshi, L.M., McComb, D.W., Dobson, C.M., and Porter, A.E. (2012). Selenium-enhanced electron microscopic imaging of different aggregate forms of a segment of the amyloid β peptide in cells. ACS Nano *6*, 4740–4747.

Michel, C.H., Kumar, S., Pinotsi, D., Tunnacliffe, A., St George-Hyslop, P., Mandelkow, E., Mandelkow, E.M., Kaminski, C.F., and Kaminski Schierle, G.S. (2014). Extracellular monomeric tau protein is sufficient to initiate the spread of tau protein pathology. J. Biol. Chem. *289*, 956–967.

Narayan, P., Orte, A., Clarke, R.W., Bolognesi, B., Hook, S., Ganzinger, K.A., Meehan, S., Wilson, M.R., Dobson, C.M., and Klenerman, D. (2012). The extracellular chaperone clusterin sequesters oligomeric forms of the amyloid-β(1-40) peptide. Nat. Struct. Mol. Biol. *19*, 79–83.

Pinotsi, D., Buell, A.K., Dobson, C.M., Kaminski Schierle, G.S., and Kaminski, C.F. (2013). A label-free, quantitative assay of amyloid fibril growth based on intrinsic fluorescence. ChemBioChem *14*, 846–850.

Pinotsi, D., Buell, A.K., Galvagnion, C., Dobson, C.M., Kaminski Schierle, G.S., and Kaminski, C.F. (2014). Direct observation of heterogeneous amyloid fibril growth kinetics via two-color super-resolution microscopy. Nano Lett. *14*, 339–345.

Rees, E., Erdelyi, M., Pinotsi, D., Knight, A., Metcalf, D., and Kaminski, C. (2012). Blind assessment of localisation microscope image resolution. Optical Nanoscopy *1*, 12.

Roberti, M.J., Bertoncini, C.W., Klement, R., Jares-Erijman, E.A., and Jovin, T.M. (2007). Fluorescence imaging of amyloid formation in living cells by a functional, tetracysteine-tagged α-synuclein. Nat. Methods *4*, 345–351.

Schneider, C.A., Rasband, W.S., and Eliceiri, K.W. (2012). NIH Image to ImageJ: 25 years of image analysis. Nat. Methods *9*, 671–675.

Su, Y., and Chang, P.-T. (2001). Acidic pH promotes the formation of toxic fibrils from β-amyloid peptide. Brain Res. *893*, 287–291.

Takahashi, R.H., Almeida, C.G., Kearney, P.F., Yu, F., Lin, M.T., Milner, T.A., and Gouras, G.K. (2004). Oligomerization of Alzheimer's β-amyloid within processes and synapses of cultured neurons and brain. J. Neurosci. *24*, 3592–3599.

Thompson, R.E., Larson, D.R., and Webb, W.W. (2002). Precise nanometer localization analysis for individual fluorescent probes. Biophys. J. *82*, 2775–2783.

Tokunaga, M., Imamoto, N., and Sakata-Sogawa, K. (2008). Highly inclined thin illumination enables clear single-molecule imaging in cells. Nat. Methods *5*, 159–161.

Walsh, D.M., Tseng, B.P., Rydel, R.E., Podlisny, M.B., and Selkoe, D.J. (2000). The oligomerization of amyloid β-protein begins intracellularly in cells derived from human brain. Biochemistry *39*, 10831–10839.

Wolter, S., Schüttpelz, M., Tscherepanow, M., van de Linde, S., Heilemann, M., and Sauer, M. (2010). Real-time computation of subdiffraction-resolution fluorescence images. J. Microsc. *237*, 12–22.

Wood, S.J., Maleeff, B., Hart, T., and Wetzel, R. (1996). Physical, morphological and functional differences between pH 5.8 and 7.4 aggregates of the Alzheimer's amyloid peptide Aβ. J. Mol. Biol. *256*, 870–877.

Chemistry & Biology, Volume *21*

Supplemental Information

Direct Observations of Amyloid β Self-Assembly in Live Cells Provide Insights into Differences in the Kinetics of Aβ(1–40) and Aβ(1–42) Aggregation

Elin K. Esbjörner, Fiona Chan, Eric Rees, Miklos Erdelyi, Leila M. Luheshi, Carlos W. Bertoncini, Clemens F. Kaminski, Christopher M. Dobson, and Gabriele S. Kaminski Schierle

I. Supplementary figures

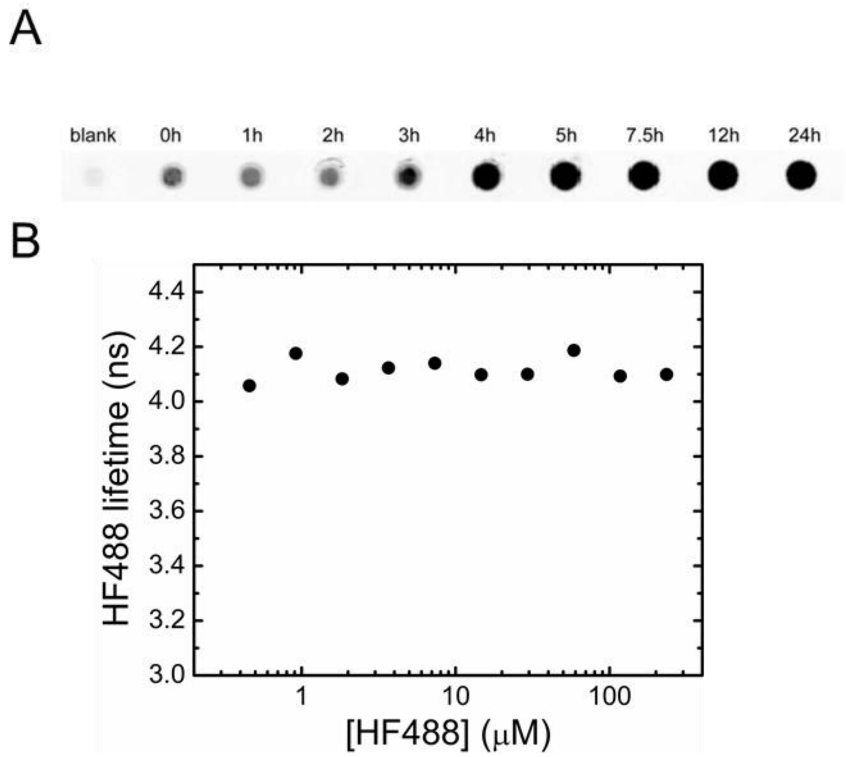

Figure S1 (related to Figure 1). *Fibril formation of HF488-labelled Aβ(1-42) peptides and fluorescence lifetimes of the HF488 dye in vitro.*

(A) Dot blot analysis of LOC immunoreactive fibrillar amyloid species in a solution of HF488-Aβ(1-42) peptide in 50 mM sodium phosphate buffer (pH 7.4) incubated in shaking condition (300 rpm orbital shaking) at ambient temperature. A densiometric analysis of this blot is shown in Fig. 1A of the main text. (B) Fluorescence lifetime of the unconjugated HF488 dye as a function of dye concentration. Note the logarithmic scale on the X-axis. The fluorescence lifetimes were measured on the FLIM microscope described in the experimental section but with the detection operated in single mode, which corresponds to a bulk measurement. The HF488 dye was suspended in 50 mM phosphate buffer, pH 7.4. The graph shows that the fluorescence lifetimes remain constant over at least two orders of magnitude higher concentration than what was used in this study to treat live cells. This shows that the HF488 fluorescence lifetime will not change merely due to the increased concentration of peptide accumulation within the cell.

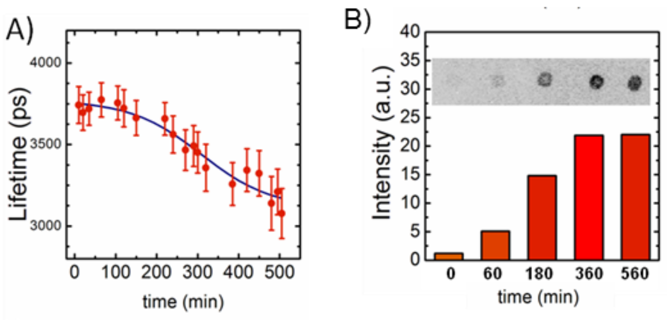

Figure S2 (related to Figure 2). *Fibril formation of HF488-labelled Aβ(1-40) in vitro.*

(A) Average fluorescence lifetime (±SD) as a function of time in an aggregating sample containing 5 μM HF488-Aβ(1-40) in 50 mM sodium phosphate buffer (pH 7.4). The data were extracted from an extended number of images from the series shown in Figure 2 of the main text. The sigmoidal curve is a fit to data and intended to guide the eye. (B) Dot blot analysis of the appearance of LOC immunoreactive fibrillar amyloid species in a sample of HF488-Aβ(1-40) peptide in 50 mM sodium phosphate buffer (pH 7.4) together with densiometric analysis of the blot showing the progressive increase in fibrillar species as a function of time.

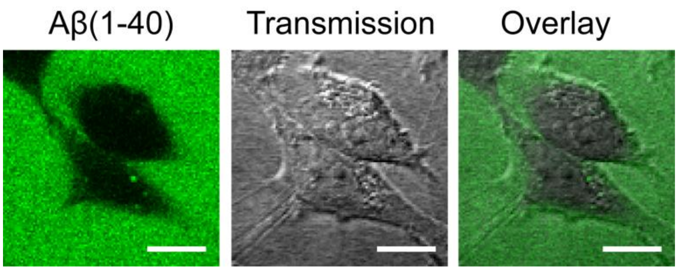

Figure S3 (related to Figure 3). *Cellular uptake of ^{HF488}Aβ(1-40) after 10 minutes of incubation.*

Fluorescence (left panel), transmission (middle panel) and overlay (right panel) confocal images showing SH-SY5Y cells treated with 500 nM ^{HF488}Aβ(1-40) for 10 minutes. The punctuate staining indicated vesicular uptake. Z-scanning was performed to ensure that the fluorescence emanated from peptide localised within the cell body. The scale bars are 10 μm.

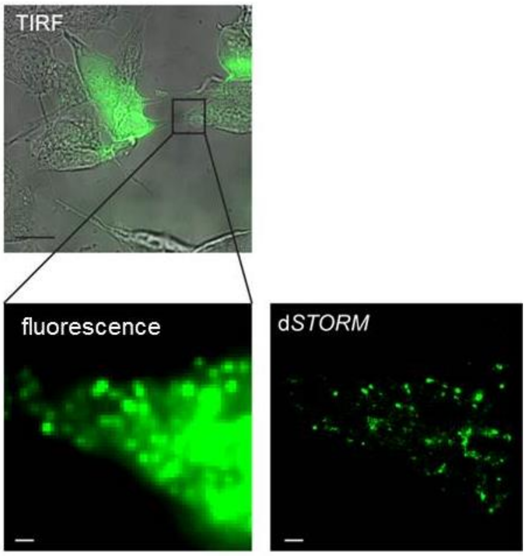

Figure S4 (related to Figure 5). *Improvement in resolution from direct stochastic optical reconstruction microscopy (dSTORM).*

Top panel: Total internal reflection (TIRF) image of a cell containing ^{HF647}Aβ(1-42). The image was acquired in highly inclined mode to access an image plane from within the cell. The zoom in depicts the area in the sample over which images for *d*STORM were acquired. Lower panels: The left panel shows the unresolved fluorescence image of the zoom in area depicted in the TIRF image and the right panel the resolved *d*STORM image. The scale bars in all images are 1 µm.

II. Supplementary Tables

Table S1 (related to Figure 4). Statistical analysis of the difference of the mean value of the intracellular lifetime of ^{HF488}Aβ(1-40) and ^{HF488}Aβ(1-42) compared to the extracellular lifetime. N denotes number of independent observations at each time point. P-values were computed in GraphPad Prism using the Fisher's Least Significant Difference (LSD) test (* $p<0.05$; ** $p<0.01$; *** $p<0.001$; **** $p<0.0001$).

	^{HF488}Aβ(1-40)			^{HF488}Aβ(1-42)		
	N	P Value	Significant	N	P Value	Significant
E.C. - 1h	2	0.55	ns	2	0.24	ns
E.C. - 3h	3	0.98	ns	3	0.12	ns
E.C. - 6h	3	0.98	ns	3	0.0028	**
E.C. - 24h	7	0.076	ns	6	< 0.0001	****
E.C. - 48h	9	0.0032	****	8	< 0.0001	****

Table S2 (related to Figure 4). Statistical analysis of the difference in intracellular lifetime between ^{HF488}Aβ(1-40) and ^{HF488}Aβ(1-42) at indicated time points. P-values were computed in GraphPad Prism using the Fisher's Least Significant Difference (LSD) test (* $p<0.05$; ** $p<0.01$; *** $p<0.001$; **** $p<0.0001$).

	^{HF488}Aβ(1-40) vs ^{HF488}Aβ(1-42)	
	P Value	Significant
0h	0.47	
1h	0.96	ns
3h	0.37	ns
6h	0.017	*
24h	0.0007	***
48h	0.22	ns
